# DEJKMDR: miRNA-disease association prediction method based on graph convolutional network

**DOI:** 10.3389/fmed.2023.1234050

**Published:** 2023-09-12

**Authors:** Shiyuan Gao, Zhufang Kuang, Tao Duan, Lei Deng

**Affiliations:** ^1^School of Computer and Information Engineering, Central South University of Forestry and Technology, Changsha, China; ^2^School of Computer Science and Engineering, Central South University, Changsha, China

**Keywords:** miRNA, miRNA-disease, JK-net, DropEdge, graph convolutional network

## Abstract

Numerous studies have shown that miRNAs play a crucial role in the investigation of complex human diseases. Identifying the connection between miRNAs and diseases is crucial for advancing the treatment of complex diseases. However, traditional methods are frequently constrained by the small sample size and high cost, so computational simulations are urgently required to rapidly and accurately forecast the potential correlation between miRNA and disease. In this paper, the DEJKMDR, a graph convolutional network (GCN)-based miRNA-disease association prediction model is proposed. The novelty of this model lies in the fact that DEJKMDR integrates biomolecular information on miRNA and illness, including functional miRNA similarity, disease semantic similarity, and miRNA and disease similarity, according to their Gaussian interaction attribute. In order to minimize overfitting, some edges are randomly destroyed during the training phase after DropEdge has been used to regularize the edges. JK-Net, meanwhile, is employed to combine various domain scopes through the adaptive learning of nodes in various placements. The experimental results demonstrate that this strategy has superior accuracy and dependability than previous algorithms in terms of predicting an unknown miRNA-disease relationship. In a 10-fold cross-validation, the average AUC of DEJKMDR is determined to be 0.9772.

## 1. Introduction

The miRNA is an endogenous non-coding single-strand RNA molecule that regulates gene expression in a significant manner. miRNA is involved in processes such as animal and plant cell differentiation, proliferation, apoptosis, and tissue and organ formation. miRNAs also perform crucial roles in a variety of vital biological processes, as evidenced by a growing number of reports. miRNAs contribute significantly to the comprehension of life sciences. Numerous aspects of microRNAs are significant, including cellular biological processes, regulation of gene expression at the transcriptional and post-transcriptional levels, and others. Understanding could be increased and experimental costs reduced if we could identify the most probable potential miRNA-disease connections and prioritize their biological experimental validation.

miRNA serve a crucial regulatory role in a variety of life processes within the human body and are tightly linked to the occurrence and development of cancer and other diseases. Methods of computational prediction have become an essential tool for discovering new disease-related miRNAs.

On the molecular mechanisms and connections that exist between microRNAs and disease, genes and disease, etc., numerous studies have been undertaken.

With regard to the connection between miRNAs and disease, Genome Tiling Arrays were suggested for universal detection of Human Transcribed Sequences by Bertone et al. (1). The related research on circulating and extracellular vesicle-derived microRNAs as biomarkers for bone-related maladies was developed by Huber et al. (2). Additionally, Zapata-Martinez et al. (3) proposed the involvement of inflammatory microRNAs in cardiovascular pathology. The role of miroRNA-21-containing microvesicles derived from renal tubular epithelial cells in cardiac hypertrophy was developed by Di et al. (4). The role of exosomal microRNAs in central nervous system diseases was explored by Yu et al. (5). Research about the progress of microRNA-361-5p in human malignant tumor was proposed by Qi et al. (6). In cancer research, the identification of regulatory mechanisms between miRNAs and genes is fundamental. It facilitates a thorough comprehension of the molecular mechanisms underlying cancer. A strategy identifying miRNA-Gene universal and specific functional modules for cancer was proposed by Chen et al. (7). A strategy predicting miRNA-Disease Associations via Node-Level Attention Graph Auto-Encoder was conducted by Zhang et al. (8). The MSGCL, an approach that utilizes multi-view self-supervised graph-based contrastive modeling for inferring miRNA–disease associations, was recommended by Ruan et al. (9). A study to explore disease regulation by investigating microRNA-dependent modulation of gene expression in GABAergic interneurons was offered by Kołosowska et al. (10).

Regarding the relationship between chromosomes and diseases, a method investigating the role of miR-143, miR-145, and the MiR-143 host gene in cardiovascular development and illness was established by Vacante et al. (11). In addition, Lu et al. (12) investigated the MicroRNA-17’s functions as an oncogene by inhibiting Smad3 expression in carcinoma of the liver. A phenotype-driven paradigm for disease and gene prioritization via bidirectional optimum corresponding lexical commonalities was discovered by Zhai et al. (13). A disease–gene association prediction algorithm that is interpretable from commencement to completion was proposed by Li et al. (14). A model using knockouts to identify significant modifications to gene expression in multiple manipulation experiments was conducted by Zhao et al. (15).

Genomics and bioinformatics developments have assisted in the identification of microRNAs. It was additionally found that miRNAs bond with a variety of prescription drugs. For example, the SVMMDR, a prediction model of miRNAs-Drug resilience employing Support Vector Machines and Heterogeneous Network, was developed by Duan et al. (16). The SVMMDR incorporates miRNAs-drug resistance association, similarities in sequencing, chemical structure, and other parallels to derive path-based Hetesim features, and collects inclined diffusion features via restart random walk. Identifying the relationships between microRNAs and drug resistance can aid in the design of effective pharmaceuticals and drug combinations. In the meantime, the interactions between distinct RNAs may also play a role in the treatment of disease and the development of new drugs. For example, the NGCICM, a novel deep learning-based method for predicting circRNA-miRNA interactions, was proposed by Ma et al. (17). A model forecasting drug-disease associations for drug repositioning via a drug-miRNA-disease heterogeneous network was created by Chen et al. (18). The prediction of small molecule drug-miRNA associations based on GNNs and CNNs was carried out by Niu et al. (19).

There are multiple public databases that catalog the relationships between miRNAs and diseases. For example, the HMDD database was created by Huang et al. (20) to curate experiential proof confirming human miRNA and disease associations. miRNAs are a type of indispensable regulatory RNA that primarily inhibit post-transcriptional gene expression. The mTD was created by Chen et al. (21) to capture the miRNAs affecting the therapeutic effects of drugs. The microRNA–cancer association database constructed by using text analysis on scientific literature was developed by Xie et al. (22) to modulate gene expressions. The TransmiR v2.0 database was developed by Tong et al. (23) to provide an updated transcription factor-microRNA regulation. The miRTarBase 2020 was developed by Huang et al. (24) to experimentally validate microRNA–target interaction. The dbDEMC 2.0 database was created by Yang et al. (25) to provide updated information about differentially expressed miRNAs in human cancers. However, the ability to predict potential associations between known miRNAs and disease from existing data sets is limited. Owing to the fact that most biological experiments are costly and laborious, it is important to develop computational techniques for predicting possible relationships between miRNAs and disease.

There are currently studies predicting a possible link between miRNAs and disease. For example, an innovative miRNA-disease association forecasting framework applying dual walk randomization with relaunch and spatial projection pooled method was developed by Li et al. (26). A fresh structure to infer miRNA-disease link was recommended by Wang et al. (27). A three-layer heterogeneous network combined with asymmetrical random paths for miRNA-disease association prediction was developed by Yu et al. (28). Logistic profile-weighted bi-random walk was suggested by Dai et al. (29) to explore miRNA-disease associations. An amalgamated ranking algorithm and a disproportionate bi-random walk on a network with heterogeneity were developed by Yu et al. (30) to infer microRNA-disease association. Biased Random Exercises with Restart on Multilayer Hierarchical Networks was conducted by Qu et al. (31) to conduct miRNA–Disease Association prediction. Analogy incorporation of networks and inductive matrix execution for miRNA–disease association prediction was carried out by Li et al. (32). A model to estimate miRNA-disease associations using a neural network was introduced by Han et al. (33). A method to predict miRNA-disease association based on graph autoencoder and a self-attention mechanism was put forward by Gao et al. (34). A model based on Neighbor Selection Graph Attention Networks for predicting miRNA-Disease associations was provided by Zhao et al. (35). A model based on multi-view graph convolutional networks for link prediction was proposed by Li et al. (36). On the basis of a broad range of biological source data and utilizing a combination of a convolutional neural network feature extractor and a high-performance learning classifier on a range of biological source material, a high-efficiency algorithm was developed by Liu et al. (37). A miRNA Disease Association Prediction precision schema utilizing consolidated Similarity Information and Layered Autoencoders was offered by Sujamol et al. (38). The prediction method based on Network—Consistency Projection for the LncRNA-Disease Associations was developed by Li et al. (39). A ranking framework for miRNA-disease association identification was proposed by Zhang et al. (40). Yan et al. (41) proposed a method called DNRLMF-MDA to predict the miRNA-disease associations based on dynamic neighborhood regularized logistic matrix factorization. A computational framework called KBMF-MDI was developed by Lan et al. (42) to measure similarities among miRNAs while the semantic and functional information of disease are used to measure similarity among diseases. A multi-relational Graph Convolutional Network model was introduced by Peng et al. (43) to construct a miRNA-gene-disease heterogeneous network and learn feature embedding for miRNAs and disease through a multi-relational graph convolutional network model.

Although there are some instruments for forecasting the miRNA-disease association, these cannot optimally fuse heterogeneous information and strengthen the reliability of prediction by conducting adaptive learning. In addition, the accuracy and performance of these methods need to be improved. To solve the aforementioned problems, a miRNA-disease association prediction model, DEJKMDR, based on graph convolution is proposed in this paper. DEJKMDR incorporates biomolecular information of miR11NA and disease, such as the functional similarity of miRNA, the semantic similarity of disease, and the similarity of Gaussian interaction properties of miRNA and disease. The DEJKMDR is used to predict potential miRNAs-disease associations. Our method’s contribution consists primarily of the following elements:

1. The DEJKMDR employs SNFS to incorporate various types of biomolecule data signatures.
2. During training, the DEJKMDR deletes random edges of the adjacency matrix, increasing the diversity of input sample data and reducing overfitting.
3. The DEJKMDR utilizes JK-Net to integrate the node representations of all previous layers into the final layer and to learn different order representations of various subgraph structures. By integrating all representations from previous layers, it eliminates the issue of graph convolution’s excessive smoothing.
4. The DEJKMDR method boosts the accuracy of predictions and has the finest AUC values among the current ones.

## 2. Materials and methods

First, statistics regarding miRNA-disease associations are accessed from the HMDD v3.2 database (20). A total of 1,206 miRNAs, 893 diseases, and 35,547 miRNAs associated with diseases are included. The miRNA and disease data used in this paper are displayed in Tables 1, 2. Secondly, according to the verified miRNA-disease association, the deweighting and equalization process is carried out to obtain the miRNA-disease association network association matrix A, which is depicted by Formula (1):


(1) $$A\left(m_{i},d_{j}\right)=\{\begin{array}{c} 1\;\mathrm{miRNA}\;\mathrm{is}\;\mathrm{associated}\;\mathrm{with}\;\mathrm{disease}\;\\ 0\;\mathrm{miRNA}\;\mathrm{is}\;\mathrm{not}\;\mathrm{associated}\;\mathrm{with}\;\mathrm{disease}\; \end{array}$$

where A( $m_{i},d_{j}$ ) = 1 represents the miRNA *m*_i_ linked with disease *d*_j_, A( $m_{i},d_{j}$ ) = 0, which exemplifies the miRNA *m*_i_ is unrelated to the disease *d*_j_.

**Table 1:** List of miRNAs.

id	miRNA name
1	hsa-mir-200b
2	hsa-mir-21
3	hsa-mir-214
…	…
1206	hsa-mir-320b-1

**Table 2:** List of disease.

id	The disease’s name	PMID
1	Colon neoplasms	15737576
2	Breast neoplasms	16466964
…	…	
893	Placenta cancer	29805755

### 2.1. DEJKMDR algorithm framework

**Figure 1:**
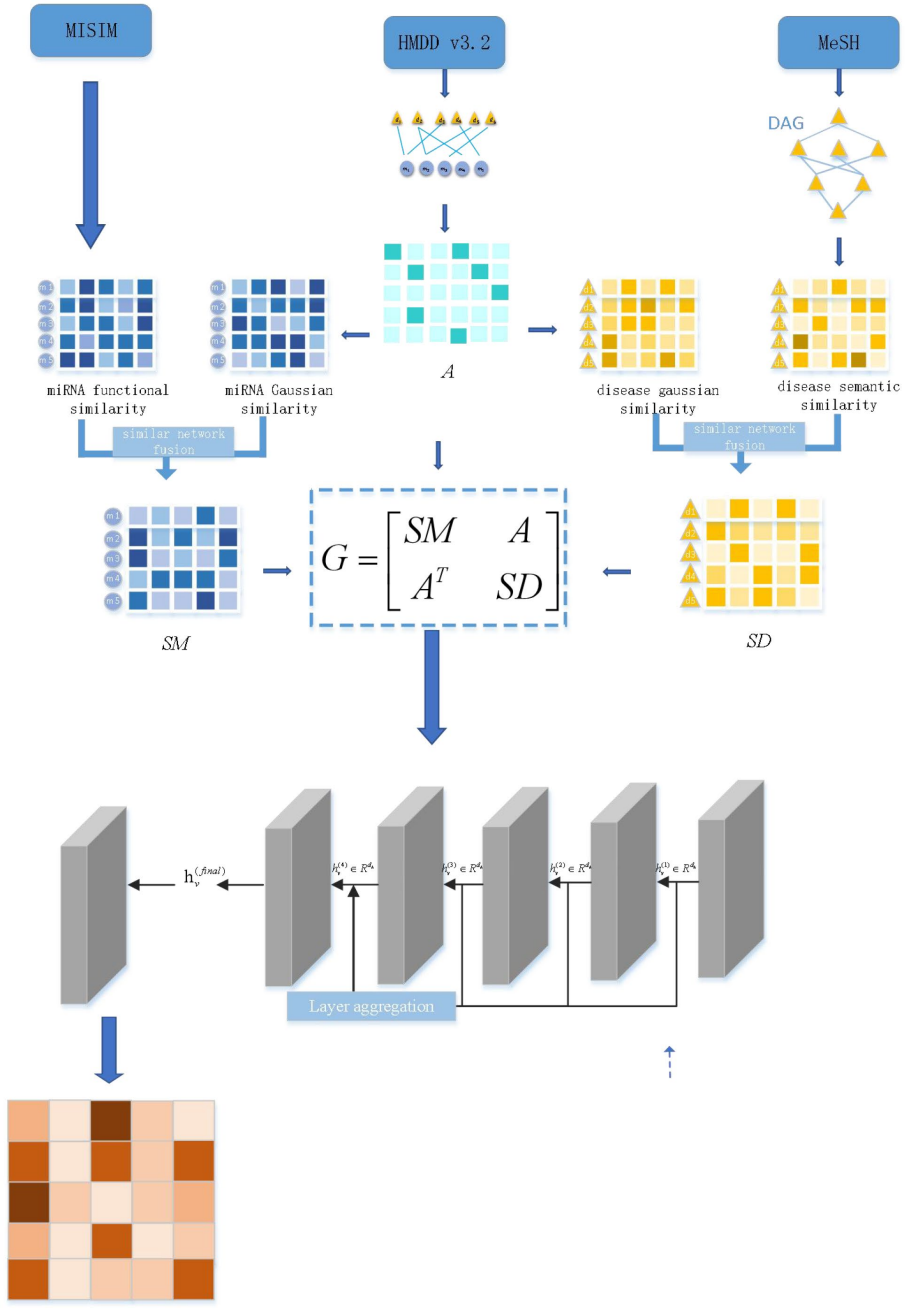
Flowchart of DEJKMDR.

Figure 1 depicts the DEJKMDR flowchart. DEJKMDR primarily consists of the following actions:

1. The miRNA-disease correlation set and mirNa-disease correlation matrix A are created, respectively, by deleting duplicate data from the miRNA-disease correlation data set retrieved from the public database HMDD v3.2 (20).
2. The SSD and FSM matrices, which stand for the semantic and operational similarity matrices, are computed, respectively.
3. The disease Gaussian interaction variable resemblance matrix GSD and the ring-shaped miRNA Gaussian relation attribute similarity matrix GSM are computed.
4. Similarity network fusion is utilized to generate disease similarity matrix SD on the basis of SSD and GSD, and similarly, miRNA similarity matrix SM is formed centered on FSM and GSM.
5. Three subnets have been implemented to build a global heterogeneous network: the miRNA-disease association network association matrix A, the miRNA similarity matrix SM, and the disease resemblance matrix SD. DropEdge is the tool for regularizing edges in heterogeneous networks to minimize overfitting by deleting some edges at random.
6. JK-Net is used to get the final predicted scores.

### 2.2. Calculation of similarity matrix

The calculation of the similarity matrix is explained in this section. This comprises calculating the disease’s semantic similarity matrix, the miRNA’s sequence similarity, and the kernel resemblance matrix for the disease’s Gaussian interaction attribute, and then creating the miRNA and disease’s final comprehensive similarity matrix.

#### 2.2.1. Disease semantic similarity matrix

This section considers the semantic similarity of disease from two aspects. Firstly, diseases’ semantic correspondence is calculated utilizing the Medical Subject Headings database (44). In this approach, directed acyclic graphs (DAGs) are applied to represent disease data structures.

**Figure 2:**
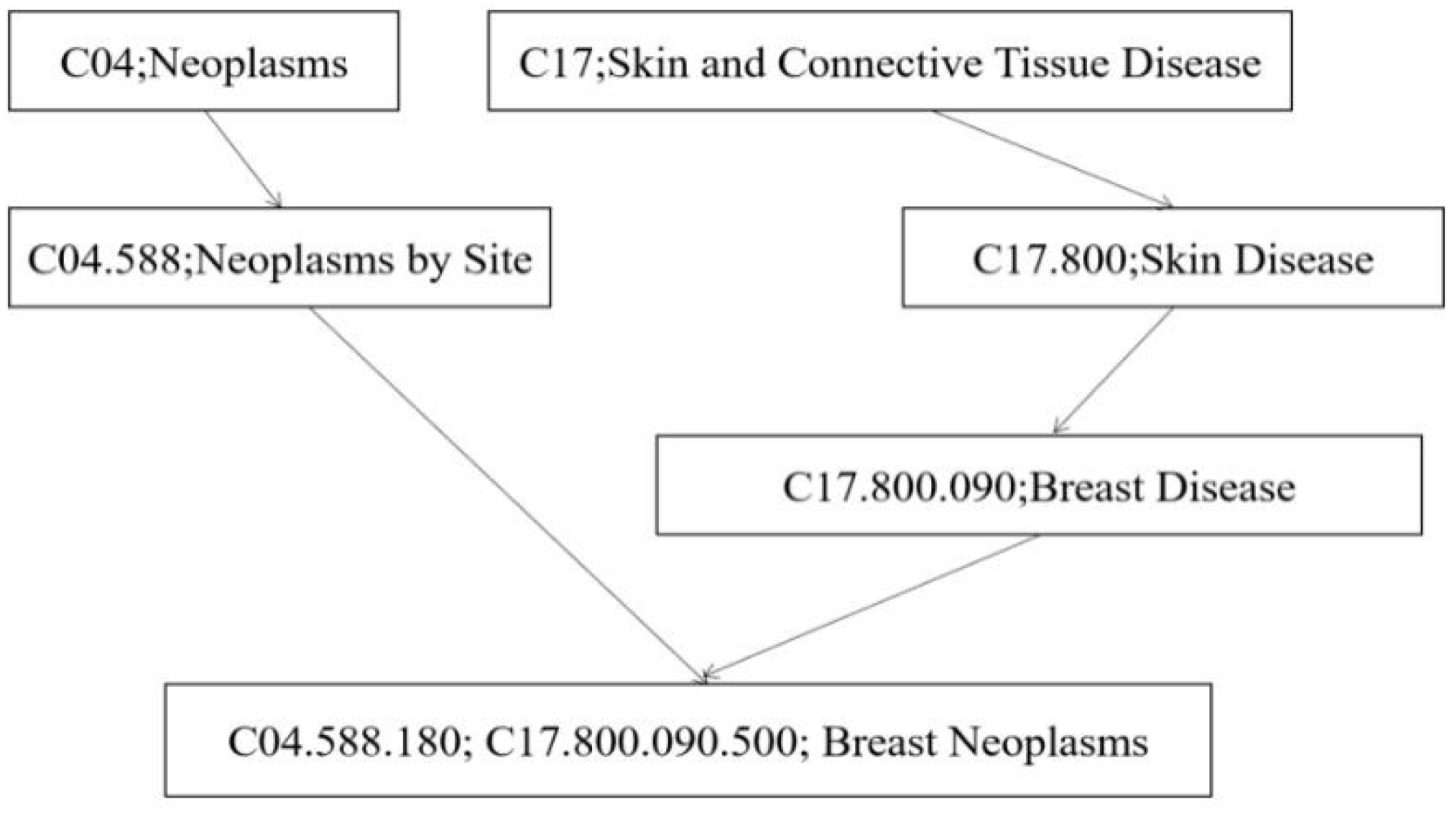
The DAG of breast neoplasms.

For example, a disease directed acyclic graph could be shown as DAG [*d*(*i*)] = {*d*(*i*),T[*d*(i)], E[*d*(*i*)]}.Here, T[*d*(*i*)] represents the ancestor node set of disease *d*(*i*), and E[*d*(*i*)] represents the edge set from the ancestor node to disease *d*(*i*). This is shown in the following Figure 2. *d*(*i*) represents Breast Neoplasms, T[*d*(*i*)] are Breast Disease，Neoplasms by Site，Skin Disease，Neoplasms，and Skin and Connective Tissue Disease. From this, the contribution of disease *d*(*n*) to the lexical measurement of disease *d*(*i*) in DAG [*d*(*i*)] can be calculated, where *n* is the other diseases within T[*d*(*i*)].


(2) $$D1_{d\left(i\right)}\left(d\left(n\right)\right)=\{\begin{array}{c} 1,\mathrm{if}\;d\left(n\right)=d\left(i\right)\\ \begin{array}{l} \max\{\Delta \times D1_{d\left(i\right)}\left(d{\left(n\right)}^{\prime }\right)|d{\left(n\right)}^{\prime }\\ \in \mathrm{children}\;\mathrm{of}\;d\left(n\right)\},\mathrm{if}\;d\left(n\right)\neq d\left(i\right) \end{array} \end{array}$$

Where Δ symbolizes the contribution factor for semantics, which will be modified to 0.5. This is shown in reference 39. Consequently, the semantic value of disease *d*(*i*) is derived as follows:


(3) $$SSV1\left(d\left(i\right)\right)=\underset{d\left(n\right)\in T\left(d\left(i\right)\right)}{\sum }D1_{d\left(i\right)}\left(d\left(n\right)\right)$$

Finally, the semantic similarity scores between disease *d*(*i*) and *d*(*j*) are computed:


(4) $$SSD1\left(d\left(i\right),d\left(j\right)\right)=\frac{\sum_{d\left(n\right)\in T\left(d\left(i\right)\right)\cap T\left(d\left(j\right)\right)}\left(\begin{array}{l} D1_{d\left(i\right)}\left(d\left(n\right)\right)\\ +D1_{d\left(j\right)}\left(d\left(n\right)\right) \end{array}\right)}{SSV1\left(d\left(i\right)\right)+SSV1\left(d\left(j\right)\right)}$$

Secondly, to calculate semantic similarity between two diseases, it is also necessary to weigh the number of occurrences of the same disease in distinct DAGs. Since diseases in different layers of the same DAG also have different semantic contribution values of diseases, from this perspective, some specific diseases may contribute more to disease *d*(*i*). Based on this theory, the semantic value contribution of disease *d*(*n*) to *d*(*i*) is shown as follows:


(5) $$D2_{d\left(i\right)}\left(d\left(n\right)\right)=-\log\left(\frac{\mathrm{the}\;\mathrm{number}\;\mathrm{of}\;\mathrm{DAGs}\;\mathrm{including}\;d\left(n\right)\;}{\mathrm{the}\;\mathrm{number}\;\mathrm{of}\;\mathrm{disease}}\right)$$

Then the logical rating of disease *d*(*i*) and the semantic similarity of disease *d*(*i*) and *d*(*i*) are obtained as follows:


(6) $$SSV2\left(d\left(i\right)\right)=\underset{d\left(n\right)\in T\left(d\left(i\right)\right)}{\sum }D2_{d\left(i\right)}\left(d\left(n\right)\right)$$

(7) $$SSD2\left(d\left(i\right),d\left(j\right)\right)=\frac{\sum_{d\left(n\right)\in T\left(d\left(i\right)\right)\cap T\left(d\left(j\right)\right)}\left(\begin{array}{l} D2_{d\left(i\right)}\left(d\left(n\right)\right)\\ +D2_{d\left(j\right)}\left(d\left(n\right)\right) \end{array}\right)}{SSV2\left(d\left(i\right)\right)+SSV2\left(d\left(j\right)\right)}$$

Finally, the semantic similarity matrix of *d*(*i*) and *d*(*i*) is obtained by combining the two semantic similarity degrees:


(8) $$SSD\left(d\left(i\right),d\left(j\right)\right)=\frac{SSD1\left(d\left(i\right),d\left(j\right)\right)+SSD2\left(d\left(i\right),d\left(j\right)\right)}{2}$$

#### 2.2.2. Matrix of miRNA functional similarity

The computation of the functional similarities of miRNA is the same as in the previous investigation by Wang et al. (45), where the practical resemblance of the two miRNAs is calculated by calculating the semantic similarity of the two disease sets associated with the two miRNAs. Assuming that miRNA *m*_i_ and miRNA *m*_j_ are associated with m and n diseases, separately, the similarity between miRNA *m*_i_ and miRNA *m*_j_ could be determined by applying equations (9) and (10) as follows:


(9) $$FSM\left(m_{i},m_{j}\right)=\frac{\begin{array}{l} \sum_{d\in D1\left(m_{j}\right)}S\left(d,D1\left(m_{i}\right)\right)\\ +\sum_{d\in D1\left(m_{i}\right)}S\left(d,D1\left(m_{j}\right)\right) \end{array}}{m+n}$$

(10) $$S\left(d,D1\left(m_{j}\right)\right)=\max_{d_{1}\in D1\left(m_{j}\right)}\left(SS\left(d,d_{1}\right)\right)$$

where $FSM\left(m_{i},m_{j}\right)$ is the miRNA functional similarity matrix, which is the maximum semantic similarity of every single illness in the disease set correlated with miRNA m_i_. $D1\left(m_{i}\right)$ a is a collection of diseases associated with miRNA *m_i_. d* is the number of diseases in disease concentration, and m is the number of diseases in disease concentration. *n* is the number of diseases in the disease cluster. $S\left(d,D1\left(m_{i}\right)\right)$ is the maximum semantic similarity of all diseases in disease set $D1\left(m_{i}\right)$ associated with miRNA m_i_ for disease *d*. *d*_1_ indicates the diseases in which D_1_(*m*_j_) diseases are concentrated. $SS(d,d_{1}$ ) represents the semantic similarity between disease d in the $D1\left(m_{i}\right)$ disease cluster and disease d_1_ in disease set $D1\left(m_{j}\right)$ . It should be noted that similarities between the disease matrix SSD and miRNA similarity matrix FSM are sparse. Therefore, the kernel similarity of Gaussian interaction attribute is further introduced to alleviate this weakness.

#### 2.2.3. Kernel similarity matrix of Gaussian interaction attribute between miRNA and disease

Both miRNA and disease show Gaussian interaction attribute kernel similarity. The similarity of Gaussian interaction kernel of disease is calculated below. Initially, the adjacency matrix is established through the associated information of miRNA and disease. The columns of the matrix represent miRNAs while the rows indicate illnesses. Additionally, applying the Gaussian kernel Function of Radial Basis Function (RBF) to the adjacency matrix yields a similar matrix to the spectral kernel of the Gaussian interaction of the disease. The Gaussian interaction spectrum of miRNA uses the same nuclear similarity calculation approach as illness. The adjacency matrix is generated by applying the associated data between miRNA and disease. The columns of the matrix represent diseases while the rows indicate miRNAs. Then, the radial basis function Gaussian kernel function is implemented to the proximity matrix to acquire a similar matrix of miRNA Gaussian interaction spectrum kernel. The specific calculation process is as follows. For A miRNA *m_i_*, its IP(*m_i_*) value is defined as row *i* of the miRNA-drug association matrix A, and the kernel similarity of Gaussian interaction attribute between every single pair of miRNA *m_i_* and miRNA *m_j_* is calculated, as shown in Equation (11):


(11) $$GSM\left(m_{i},m_{j}\right)=\exp\left(-\gamma_{m}{\left\|IP\left(m_{i}\right)-IP\left(m_{j}\right)\right\|}^{2}\right)$$

(12) $$\gamma_{m}=\gamma \prime_{m}/\left(\frac{1}{\mathrm{nm}}\overset{\mathrm{nm}}{\underset{i=1}{\sum }}{\left\|IP\left(m_{i}\right)\right\|}^{2}\right)$$

Where GSM represents the kernel similarity matrix of the Gaussian interaction attribute of miRNA. Element GSM ( $m_{i},m_{j}$ ) represents the kernel similarity of the Gaussian interaction properties of miRNA *m_i_* and miRNA *m_j_*. $\gamma_{m}$ is employed to control the bandwidth of kernel similarity of Gaussian interaction attribute. It represents the normalized Gaussian interaction attribute kernel similarity bandwidth based on the new bandwidth parameter $\gamma \prime_{m}$ . nm represents the number of miRNAs.

Likewise, based on the hypothesis that there is an association between functionally similar miRNAs and similar diseases, a Gaussian interaction attribute kernel similarity matrix GSD for diseases is constructed by using the identified miRNA-disease association network.

For a disease, its ${IP}^{\prime }\left(d_{i}\right)$ value is described as column *i* of miRNA-disease correlation matrix A. The kernel similarity of Gaussian interaction attributes between each pair of diseases is calculated, as shown in Equation (13):


(13) $$GSD\left(d_{i},d_{j}\right)=\exp\left(-\gamma_{d}{\left\|IP^{\prime }\left(d_{i}\right)-IP^{\prime }\left(d_{j}\right)\right\|}^{2}\right)$$

(14) $$\gamma_{d}=\gamma \prime_{d}/\left(\frac{1}{nd}\overset{nd}{\underset{i=1}{\sum }}{\left\|IP^{\prime }\left(d_{i}\right)\right\|}^{2}\right)$$

Where, GSD represents the kernel similarity matrix of the Gaussian connection attribute of disease.

The element $GSD\left(d_{i},d_{j}\right)$ represents kernel resemblance of the Gaussian interaction characteristic of disease $d_{i}$ and disease $d_{j}$ . $\gamma_{d}$ represents standardized Gaussian interaction kernel closeness bandwidth determined by bandwidth parameters $\gamma \prime_{d}$ . and represents the number of diseases.

### 2.3. Similar network convergence

Despite the fact that the disease semantic similarity matrix and the miRNA functional similarity network have been obtained through the aforementioned techniques, further research is warranted; owing to the paucity of valuable information, these similarity matrices are rare. In order to enrich the similarity matrix, the kernel likeness of the Gaussian interaction matrix of miRNA and the kernel resemblance of the disease engagement band are calculated according to the recognized connection between miRNA and disease. At the same time, similarity network fusion is employed for fusion. SNF is an effective method for fusion of different types of data features. SNF generates an equivalent system matrix for every possible similarity and employs the non-linear combination method relying on k-nearest neighbor to integrate two networks. For miRNA, functional similarity matrix FSM and Gaussian interaction spectrum kernel similarity matrix GSM have been obtained. First, the FSM and GSM lines are normalized to get RFSM and RGSM. After using KNN, KRFSM and KRGSM are obtained, as shown in the formulas (15) and (16).


(15) $$\mathrm{KRFSM}\left(m_{i},m_{j}\right)=\{\begin{array}{c} \frac{\mathrm{RFSM}\left(m_{i},m_{j}\right)}{\sum_{m_{k}\in N\left(m_{i}\right)}\mathrm{RFSM}\left(m_{i},m_{j}\right)}\;m_{j}\in N\left(m_{i}\right)\;\\ 0\;\mathrm{otherwise} \end{array}$$

(16) $$\mathrm{KRGSM}\left(m_{i},m_{j}\right)=\{\begin{array}{c} \frac{\mathrm{RGSM}\left(m_{i},m_{j}\right)}{\sum_{m_{k}\in N\left(m_{i}\right)}\mathrm{RGSM}\left(m_{i},m_{j}\right)}\;m_{j}\in N\left(m_{i}\right)\;\\ 0\;\mathrm{otherwise} \end{array}$$

Where *N*(*m_i_*) is the collection of K nearest neighbors of m_i_. Finally, multiple similar networks are fused using an iterative method.


(17) $$RFSM_{t}=\mathrm{KRFSM}\times RGSM_{t-1}\times \mathrm{KRFS}M^{T}，t\geq 1$$

(18) $$RGSM_{t}=\mathrm{KRGSM}\times RFSM_{t-1}\times \mathrm{KRGS}M^{T}，t\geq 1$$

Where *t* is the number of iterations. $RFSM_{0}=\mathrm{RFSM}$ and $RGSM_{0}=\mathrm{RGSM}$ . After iterating *t* times, we get the final $RFSM_{t},RGSM_{t}$ . The average sum between $RFSM_{t}\;\mathrm{and}\;RGSM_{t}$ is taken as the miRNA set similarity matrix:


(19) $$MM=\frac{RFSM_{t}+RGSM_{t}\;}{2}$$

By means of the identical method, the disease integration similarity matrix DD is obtained.

### 2.4. Model training and prediction

The miRNA similar network, disease similar network, and miRNA-disease association matrix obtained after fusion of similar networks were constructed into graph structure data and input into the JK-Net model for training to obtain a prediction model. In the training process, the random edge removal rate is set as 0.4. JK-Net uses multi-layer graph convolutional neural network for representation learning of nodes to aggregate node information in different fields and can adjust adaptively according to the position of nodes in the network and the topology structure of the graph to better represent both the local and broader traits of network nodes.

## 3. Results and discussion

### 3.1. Data sets

Part of the experimental parameters in the DEJKMDR method will be introduced in this section, and part of the parameters used by DEJKMDR are displayed in Table 3.

**Table 3:** Some experimental parameters of DEJKMDR.

Sign	Value	Definition
nl	1206	The number of miRNA
ne	893	The number of diseases
n	2099	The sum of miRNA and the disease
$\gamma \prime_{l}$	1	The Gaussian interaction properties of miRNA kernels are identical in bandwidth
$\gamma \prime_{e}$	1	The Gaussian interaction properties of disease kernels are similar in bandwidth
K	10	The nearest number of k-nearest neighbors (KNN) in similar network convergence
p	0.4	DropEdge’s deletion rate
Epochs	1000	GCN training times

### 3.2. Performance measures

#### 3.2.1. Cross validation

**Figure 3:**
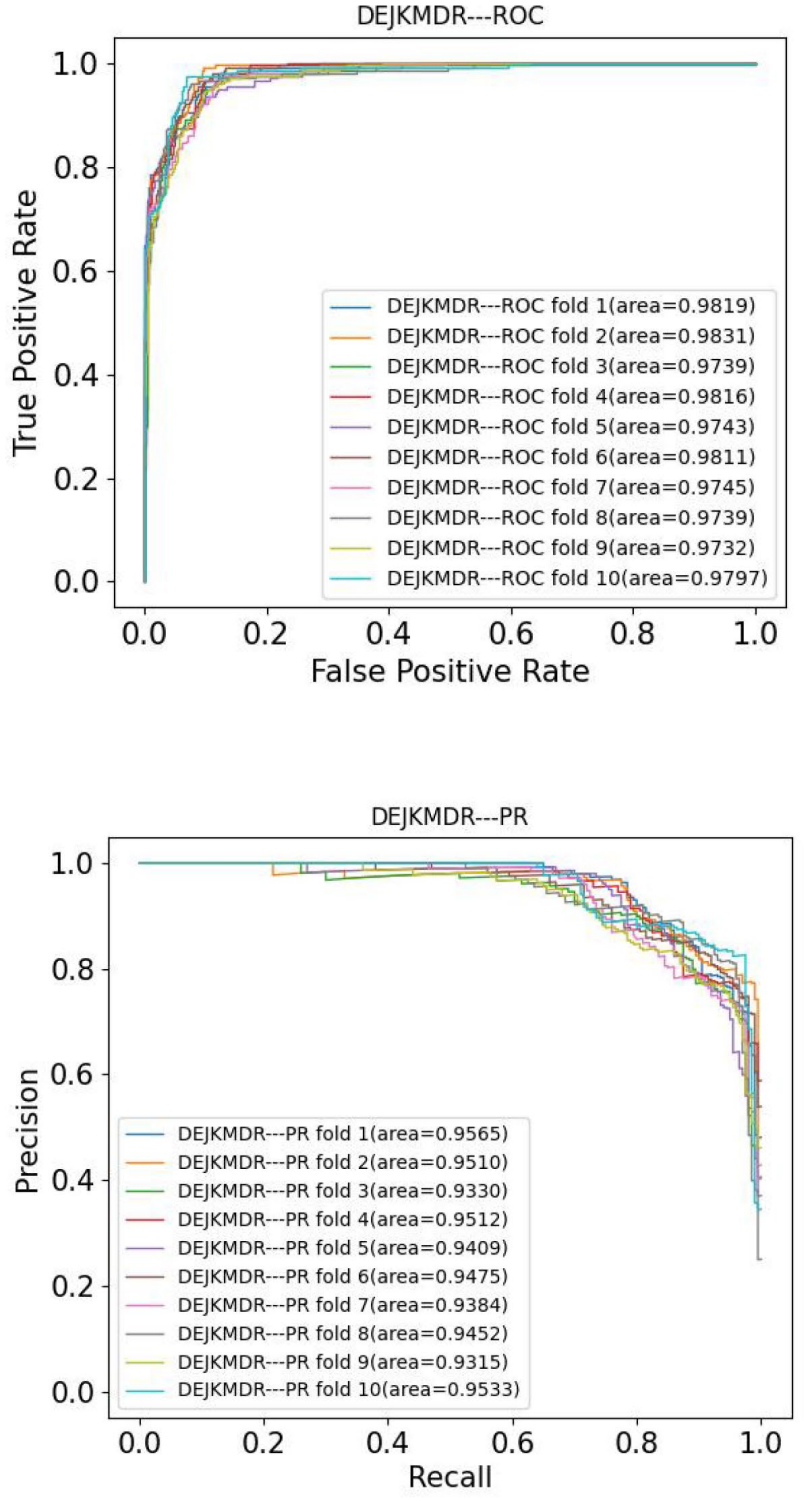
The ROC and PR curve of DEJKMDR under 10-fold cross validation.

**Figure 4:**
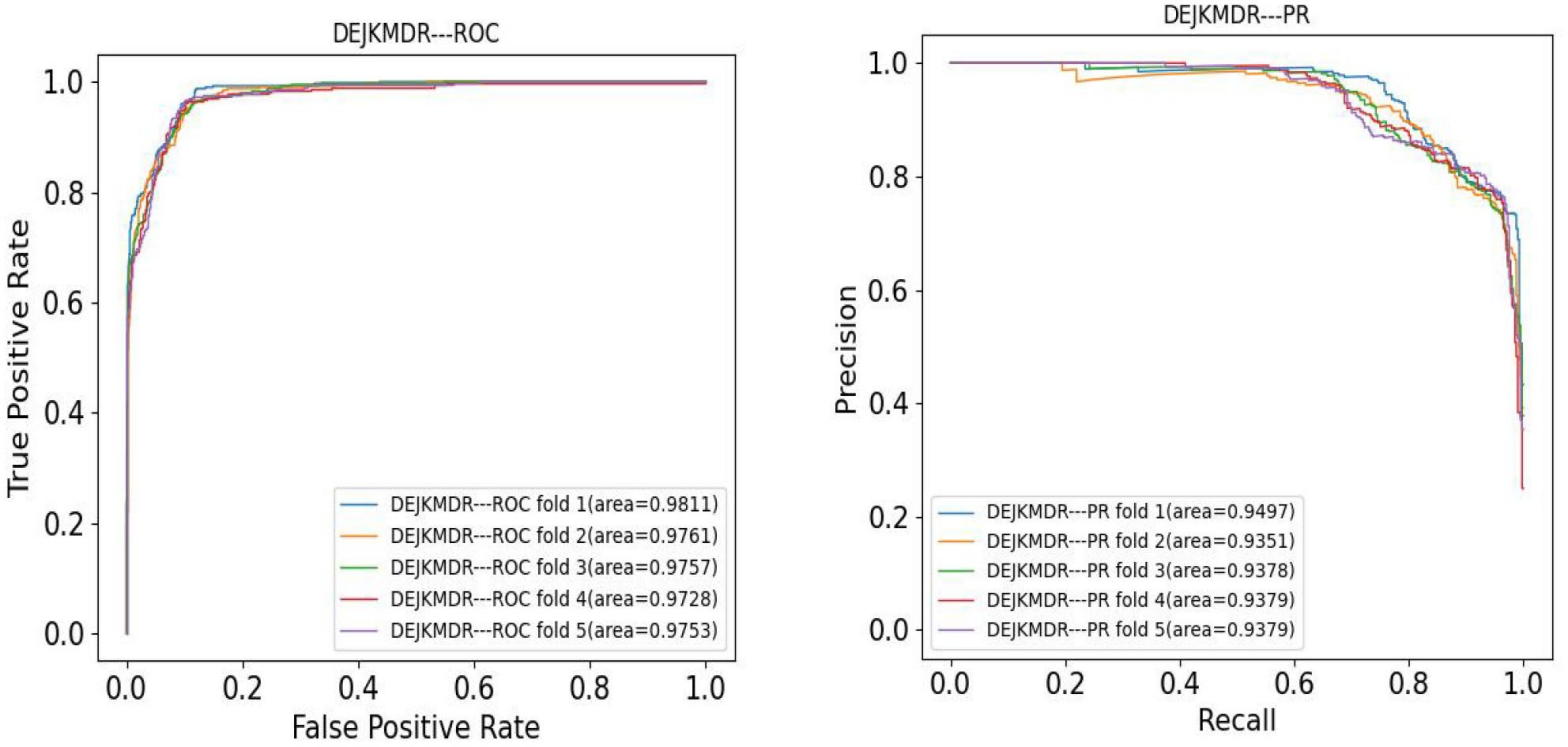
The ROC and PR curve of DEJKMDR under 5-fold cross validation.

In the aim to appraise the effectiveness of DEJKMDR about predicting miRNA-disease association, this study employs 5-fold and 10-fold cross-validation techniques. ROC and PR curves are acquired, respectively. According to Figures 3, 4, the final average AUC value of 5-fold cross-validation is 0.976193 and AUPR is 0.939682. The average AUC value and AUPR of the 10-fold cross-validation are 0.97772 and 0.944819, respectively.

#### 3.2.2. Performance comparison of different edge loss rates

**Figure 5:**
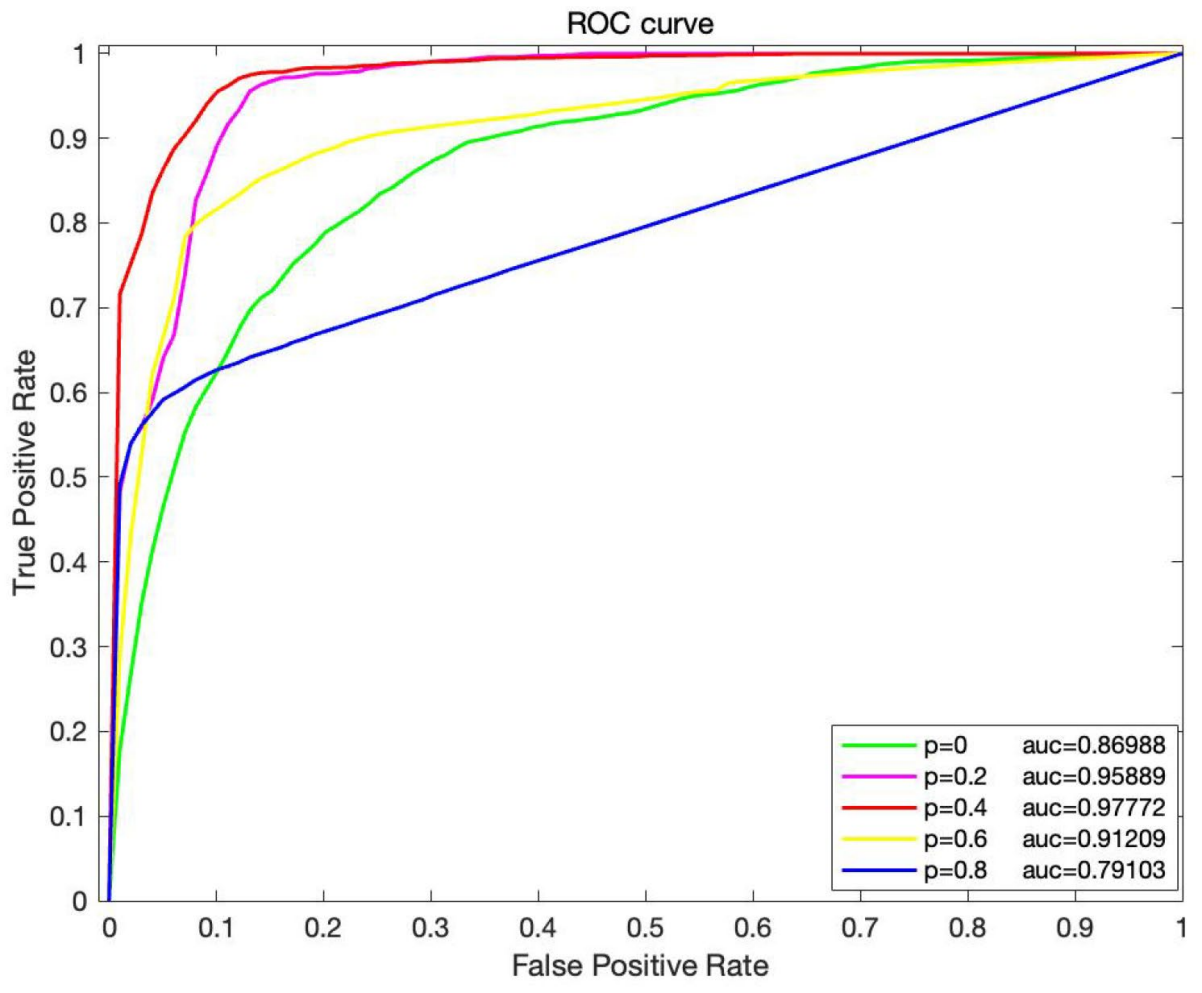
The ROC and PR curves of DEJKMDR with varied edge drop rate.

**Figure 6:**
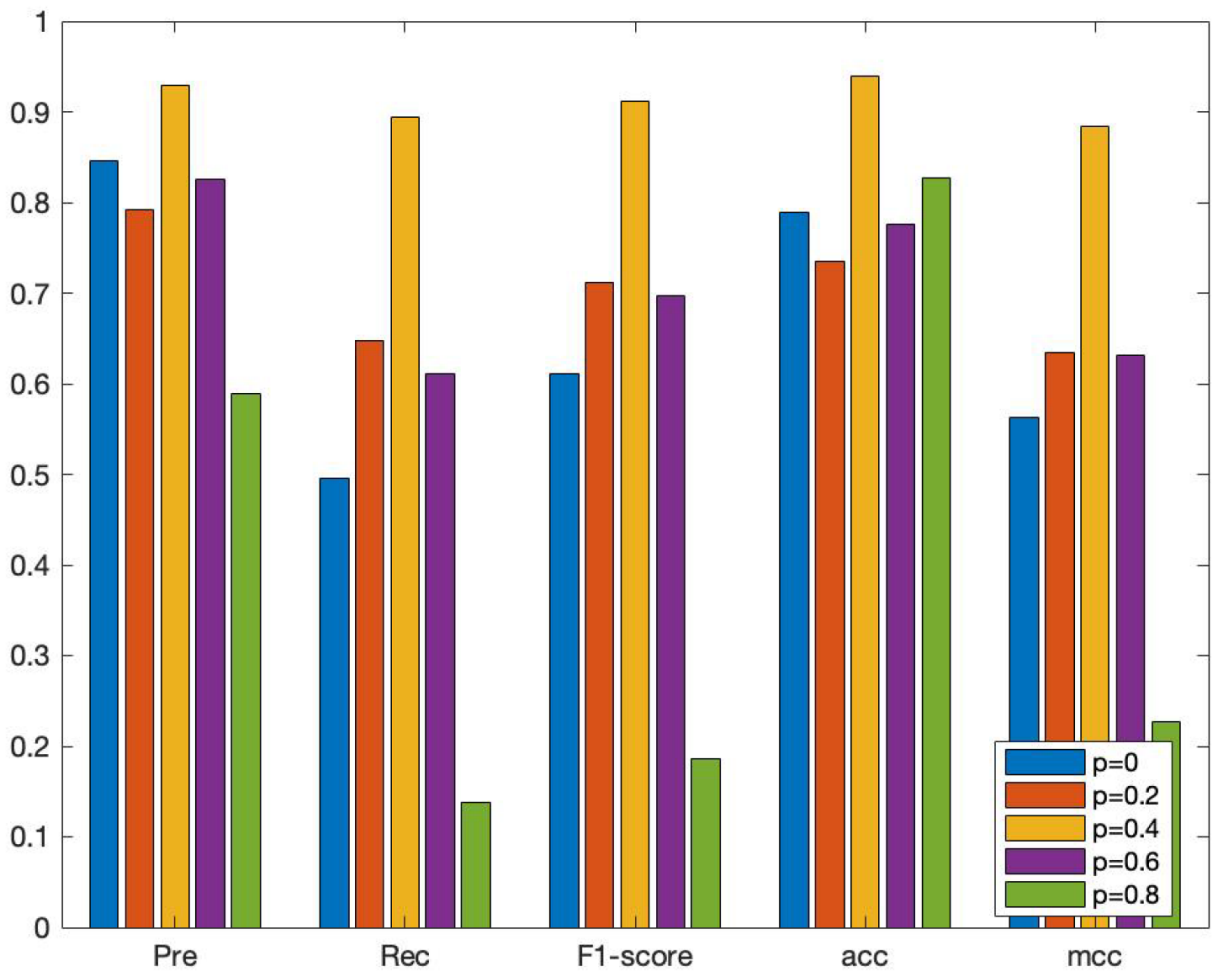
The results of the performance comparison of DEJKMDR with different edge drop rates.

To investigate the effect of various edge loss rates on the efficacy of the DropEdge method model, several groups of comparative experiments are conducted, and the edge loss rates are set as 0, 0.2, 0.4, 0.6, and 0.8, respectively. The average AUC and other performance indicators are also verified using the 10-fold crossover, as shown in Figures 5, 6. When *p* = 0, it means that the original adjacency is used as input for training. In Figure 4, it can be discovered that when *p* = 0, the AUC obtained is 0.869, and with a rise in loss rate *p*, the AUC also increases on a gradual basis. When *p* is 0.4, the ROC curve obtains the maximum AUC area and reaches a small vertex. When *p* continues to increase to 0.6 and 0.8, AUC gradually decreased, indicating that high edge loss rate would reduce model performance. Further, Figure 6 shows other performance indicators, such as accuracy, recall, and F1 scores at different edge loss rates. Similar to the AUC, these metrics also show the best performance advantage at a drop rate of 0.4. These experimental results indicate that the DropEdge strategy can considerably enhance the performance of the model, but too high edge loss rate will lead to performance degradation.

#### 3.2.3. Ablation experiment

**Figure 7:**
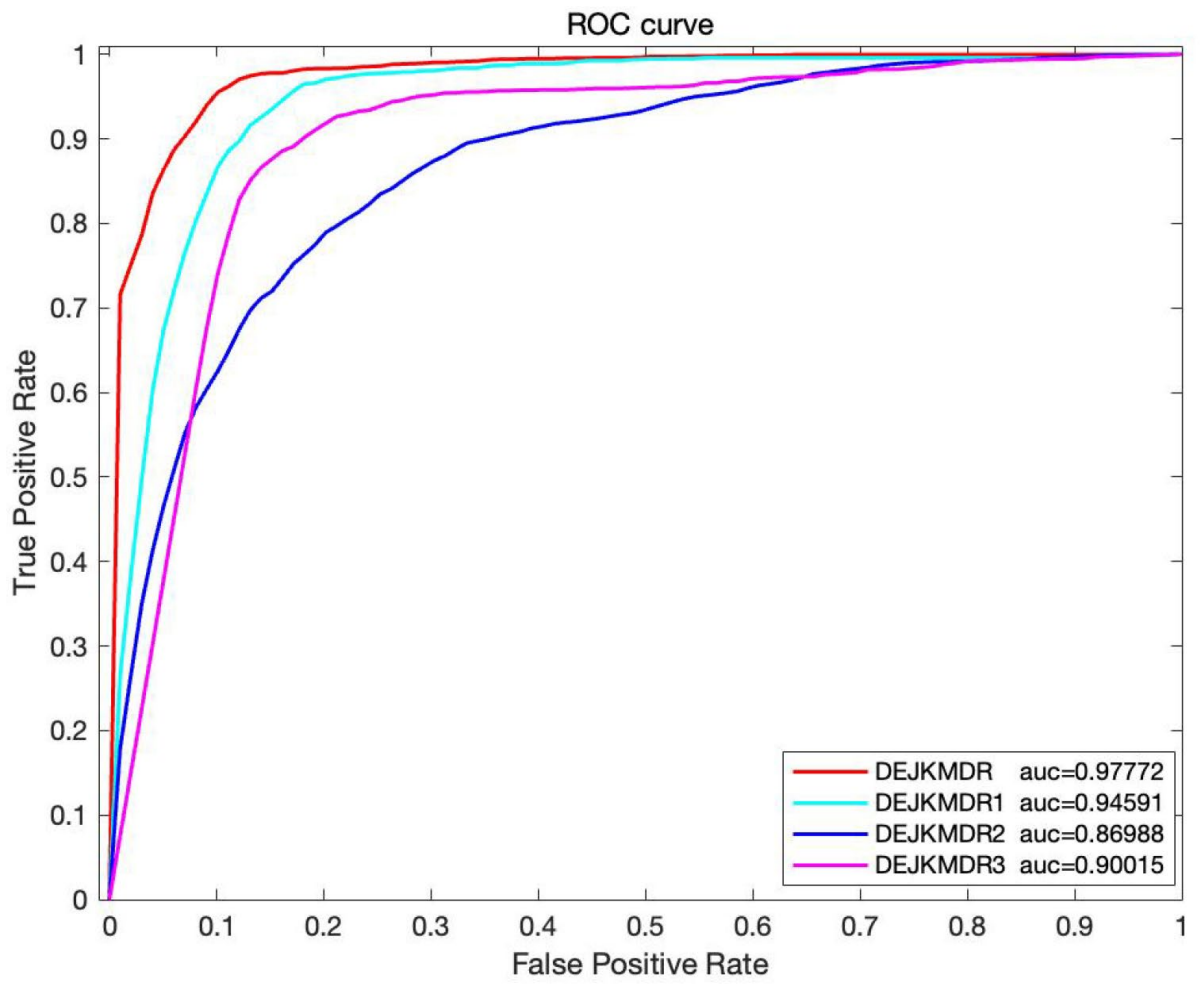
The ROC curve of Ablation study.

**Figure 8:**
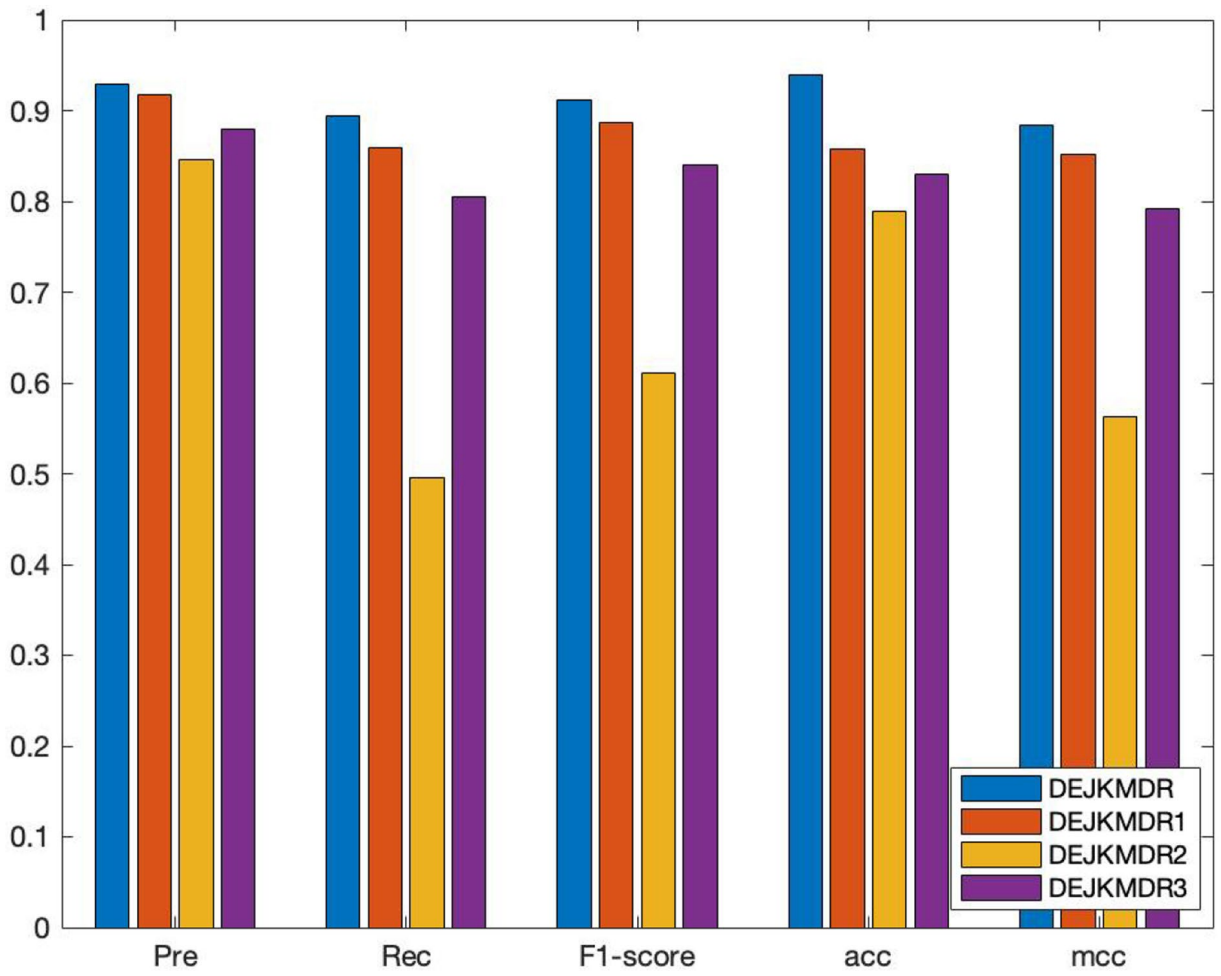
The results of the performance comparison of Ablation study.

In an effort to confirm the performance advantages of similar network fusion, random edging, and JK-Net in the model, several ablation experiments are carried out based on the proposed DEJKMDR, and several groups of comparison experiments are designed to evaluate the effectiveness of these strategies by changing the structure of the model. By means of these investigations, we can better understand the contribution and function of these methods in the model. The average AUC and other performance indexes are also obtained by using the 10-fold crossover. As shown in Figure 6, DEJKMDR in the figure is the ROC curve obtained by this model. DEJKMDR1 is the ROC curve obtained by using average value to integrate multiple similar networks, but DropEdge and JK-Net are used for prediction. DEJKMDR2 is the ROC curve obtained by this model without using DropEdge. In other words, it is the ROC curve obtained without random edge deletion operation and with other structures remaining unchanged. DEJKMDR3 is the ROC curve obtained by replacing JK-Net module in this model with ordinary graph convolution and retaining other structures for prediction. According to Figure 7, the AUC of DEJKMDR, DEJKMDR1, DEJKMDR2, and DEJKMDR3 are 0.977, 0.945, 0.869, and 0.900, respectively. Figure 8 indicates other performance indicators under these experiments. Similar to AUC, DEJKMDR has obvious advantages over other experiments in terms of accuracy and recall rate. The results show that similar network fusion, random edging, and JK-Net are used to enhance the method performance.

#### 3.2.4. Efficacy comparison with current methods

Some studies have predicted the potential association between miRNAs and disease, comparing the DEJKMDR algorithm with existing methods for predicting the relationship between miRNAs and drug susceptibility. In the experiment, three methods are selected to compare with the proposed DEJKMDR method. They are NIMGSA (32), TCRWMDA (28), and GAEMDA (36). These methods have been compared with existing methods under the same data.

##### 3.2.4.1. NIMGSA

NIMGSA is an end-to-end deep learning framework which integrates inductive matrix completion and tag propagation (32). It implements a self-attention mechanism through inductive matrix completion of two graph autoencoders, while combining inductive matrix completion and tag propagation utilizing a neural network architecture.

##### 3.2.4.2. TCRWMDA

TCRWMDA is a three-layer heterogeneous network miRNA-disease association prediction algorithm combined with non-equilibrium random walk (28). TCRWMDA operates on more than just known microRNAs associated disease and includes more data (lncRNA—microRNAs and lncRNA—disease association) to construct three distinct levels of heterogeneous network. To this is added the lncRNA as the shift of moderate spot route, allowing greater reliability between networks.

##### 3.2.4.3. GAEMDA

The GAEMDA model uses a graph-based neural network encoder consisting of a clustering operation and multi-layer perceptron, to aggregate the adjacent data from nodes, produce low-dimensional embedding of miRNA and disease nodes, and accomplish operational fusion of heterogeneous info, subsequently embedding microRNAs and disease node input bilinear decoders to identify potential connections between miRNAs and disease nodes (36).

**Figure 9:**
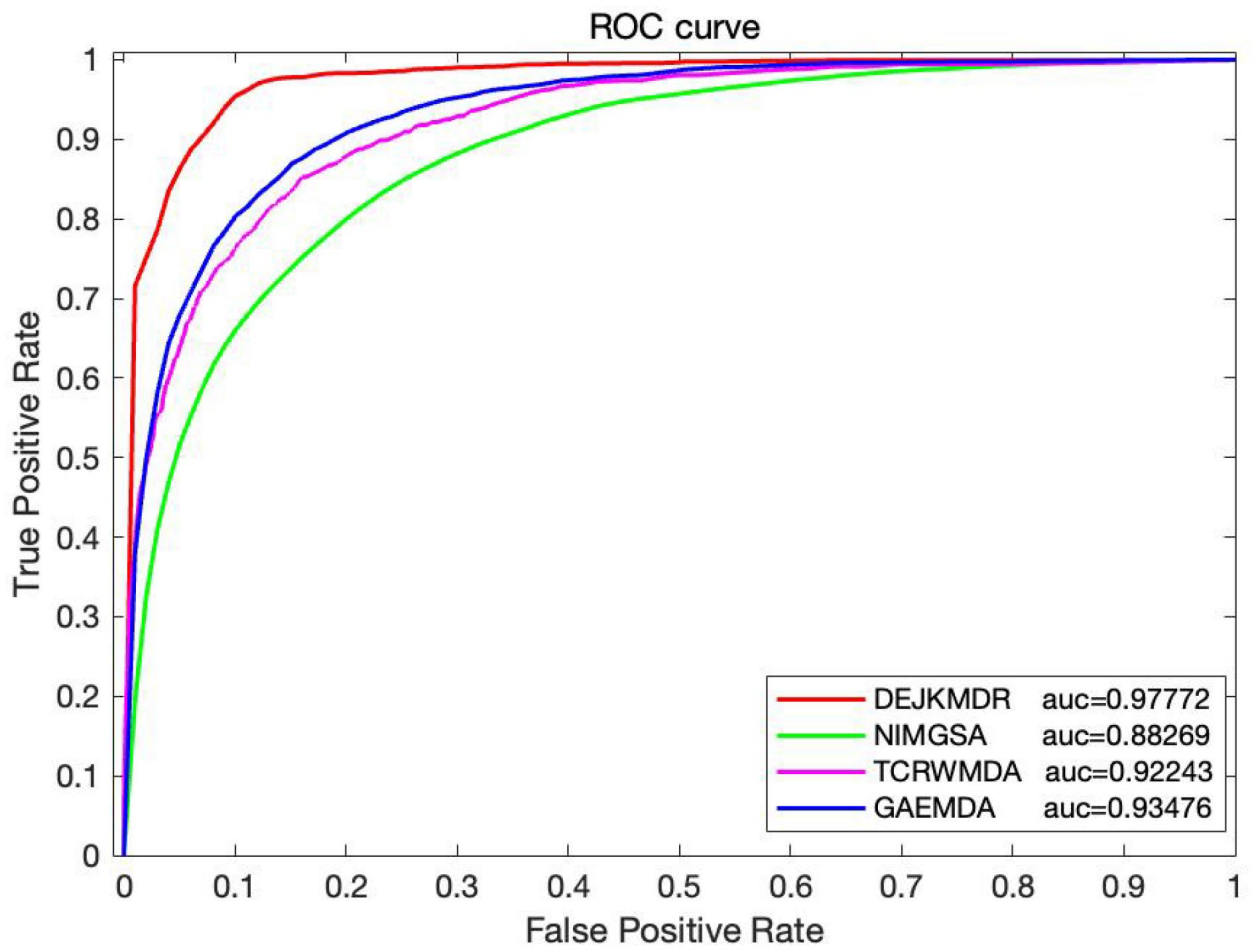
Performance comparison with existing methods.

Figure 9 displays the comparison details, showing that the DEJKMDR method outperforms the others.

The reasons are as follows: first, DEJKMDR integrates several biomolecular data types using SNF; secondly, DropEdge randomly deletes some adjacency matrix edges during training, increasing input sample data diversity and reducing overfitting. Finally, JK-Net combines the node representations of all previous layers in the last layer to learn different order representations of different subgraph structures. By combining all representations from previous layers, the problem of over-smoothing graph convolution is alleviated. All of these enable DEJKMDR to achieve better performance.

## 4. Conclusion

More and more studies have shown that the expression level of miRNAs is closely related to the occurrence and development of a variety of tumors. Predicting the association between miRNA-disease can help to identify early diagnosis protocols for the disease and prognostic observation of diseases. Therefore, in this paper, an outstanding durability technique based on heterogeneous networks for predicting the association between miRNAs and disease (DEJKMDR) is proposed. Firstly, DropEdge is used to regularize the edges in the original adjacency matrix and some edges are randomly deleted to reduce overfitting. At the same time, JK-Net is used to gather the domain information of nodes. The effect of DEJKMDR is demonstrated by 10-fold cross-validation. Compared with other current excellent prediction models, DEJKMDR is effective at predicting undocumented miRNA-disease associations because of its substantial enhancements in performance.

## Data availability statement

The original contributions presented in the study are included in the article/supplementary material, further inquiries can be directed to the corresponding author.

## Author contributions

SYG, ZFK, TD, and LD conceived this work and designed the experiments. Both SYG and ZFK conducted the experiments and gathered the data and performed the analysis. The article was composed, revised, and approved by SYG, ZFK, TD, and LD. All authors contributed to the article and approved the submitted version.

## Funding

This work was supported in part by the National Natural Science Foundation of China under Grants Nos. 62072477, 61309027, 61702562 and 61702561, the Hunan Provincial Natural Science Foundation of China under Grants No.2018JJ3888, the Hunan Key Laboratory of Intelligent Logistics Technology 2019TP1015.

## Conflict of interest

The authors declare that the research was conducted in the absence of any commercial or financial relationships that could be construed as a potential conflict of interest.

## Publisher’s note

All claims expressed in this article are solely those of the authors and do not necessarily represent those of their affiliated organizations, or those of the publisher, the editors and the reviewers. Any product that may be evaluated in this article, or claim that may be made by its manufacturer, is not guaranteed or endorsed by the publisher.
